# Supplementary material for: Disease progression modelling reveals heterogeneity in trajectories of Lewy-type α-synuclein pathology
Source: Nat Commun. 2024 Jun 15;15:5133. doi: 10.1038/s41467-024-49402-x (PMC11180185; doi:10.1038/s41467-024-49402-x)
Supplement: Supplementary file 1 — Supplementary Information [file 41467_2024_49402_MOESM1_ESM.pdf]

SUPPLEMENTARY INFORMATION

Disease progression modelling reveals heterogeneity in trajectories of Lewy-type α-synuclein pathology

Supplementary Tables

- Table S1. Demographics of the SuStaIn modelling cohort
- Table S2. Frequency of other clinicopathologic diagnoses in the SuStaIn modelling cohort
- Table S3. Lewy type α-synuclein scores of subjects assigned to SuStaIn stage 0
- Table S4. Neuropathological density scores converted to score probabilities

Supplementary Figures

- Figure S1. Model fit for up to five SuStaIn subtypes
- Figure S2. Output of the SuStaIn model applied to complete data only (*N*=701)
- Figure S3. Output of the SuStaIn model applied to re-examined OBT data
- Figure S4. Subtype assignment probability across assigned stage
- Figure S5. Distribution of stage assignment
- Figure S6. Number of subjects in stages of the Unified Staging Scheme for Lewy Body disorders according to SuStaIn stage
- Figure S7. Depigmented neurons in the substantia nigra and GBA mutation status across LB subtypes
- Figure S8. Sliding window analysis of LB subtype comparisons
- Figure S9. Total body Lewy body pathology across subtypes with a SuStaIn stage<10
- Figure S10. Example images of various LTS density scores in the olfactory bulb

SUPPLEMENTARY TABLES

Table S1. Demographics of the SuStaIn modelling cohort

	SuStaIn modelling cohort
N **(%)**	814
**Age at death (years)**	81.8 (7.7)
**Female, n (%)**	317 (38.9)
**Education, years**[a]	14.9 (2.8)
APOE **ε4 carrier, n (%)**[b]	340 (41.8)
**MMSE score**[c]	17.0 (9.6)
**Δt MMSE and death (months)**	21.8 (21.5)
**Clinicopathological diagnosis**	
**AD, n (%)**	285 (35.0)
**PD, n (%)**	168 (20.6)
**DLB, n (%)**	19 (2.3)
**Mixed AD and PD, n (%)**	78 (9.6)
**Mixed AD and DLB, n (%)**	141 (17.3)
**ILDB, n (%)**	90 (11.1)
**Other**[d]**, n (%)**	33 (4.1)
**Total Lewy body density**[e]	18.1 (11.6)
**Total plaque load**[f]	9.74 (5.7)
**Total neurofibrillary load**[g]	8.78 (4.7)
Post-mortem **interval (hours)**	5.43 (9.7)

Data are shown as mean (standard deviation) unless otherwise specified.

[a] *n*=659.

[b] *n*=807.

[c] *n*=672.

[d] Other indicates the absence of an AD or Lewy body disorder diagnosis.

[e] Sum of the regional density scores (range 0-40), *n*=701.

[f] *n*=804.

[g] *n*=801.

AD = Alzheimer's disease; DLB = dementia with Lewy bodies; ILDB = incidental Lewy body disease; PD = Parkinson's disease.

Table S2. Frequency of other clinicopathologic diagnoses in the SuStaIn modelling cohort

	**SuStaIn modelling cohort with any other clinicopathological diagnosis (*n*=33)**
**Argyrophilic grain dementia**	13 (39.4)
**Progressive supranuclear palsy**	11 (33.3)
**Cerebral amyloid angiopathy**	9 (27.3)
**Vascular dementia**	7 (21.2)
**Hippocampal sclerosis**	5 (15.2)
**FTLD-TDP**[a]	4 (12.1)
**Corticobasal degeneration**	2 (6.1)
**Pick's disease**	2 (6.1)
**Dementia lacking distinctive histology**	1 (3.0)
**Motor neuron disease**	1 (3.0)
**Huntington's disease**	1 (3.0)

Other is defined as the absence of a Lewy body disease or Alzheimer's disease diagnosis. Data are shown as number (percentage).

[a] not assessed for *n*=15 (45.5%).

FTLD-TDP = Frontotemporal lobar degeneration with TPD-43-immunoreactive pathology.

Table S3. Lewy type α-synuclein scores of subjects assigned to SuStaIn stage 0

OBT	Medulla	Pons	Substantia nigra	Amygdala	Transentorhinal cortex	Cingulate cortex	Temporal cortex	Frontal cortex	Parietal cortex
0	1	0	0	1	0	0	0	0	0
0	0	0	0	1	0	0	0	0	0
0	1	0	0	0	0	0	0	0	0
0	1	0	0	0	0	0	0	0	0
0	1	0	0	0	0	0	0	0	NA
0	0	0	1	1	0	0	0	0	0
0	0	0	0	1	0	1	0	0	0
0	1	0	0	0	0	0	0	0	0
0	1	0	0	0	0	0	0	0	0
0	0	0	0	2	0	0	0	0	0
NA	0	1	NA	0	0	0	0	0	0
0	0	0	0	1	0	0	1	0	0
0	0	0	NA	1	1	0	0	0	0

Description in terms of Lewy type α-synuclein density scores of subjects assigned to SuStaIn stage 0. Scores range from 0 to 4, with higher scores indicating more severe pathology.

Table S4. Neuropathological density scores converted to score probabilities

		Score Probability				
		0	1	2	3	4
Neuropathological density score	0	0.88	0.12	2.95×10^{-4}	1.34×10^{-8}	1.12×10^{-14}
	1	0.11	0.79	0.11	2.64×10^{-4}	1.20×10^{-8}
	2	2.64×10^{-4}	0.11	0.79	0.11	2.64×10^{-4}
	3	1.20×10^{-8}	2.64×10^{-4}	0.11	0.79	0.11
	4	1.12×10^{-14}	1.34×10^{-8}	2.95×10^{-4}	0.12	0.88
	Missing	0.20	0.20	0.20	0.20	0.20

Figure S1. Model fit for up to five SuStaIn subtypes

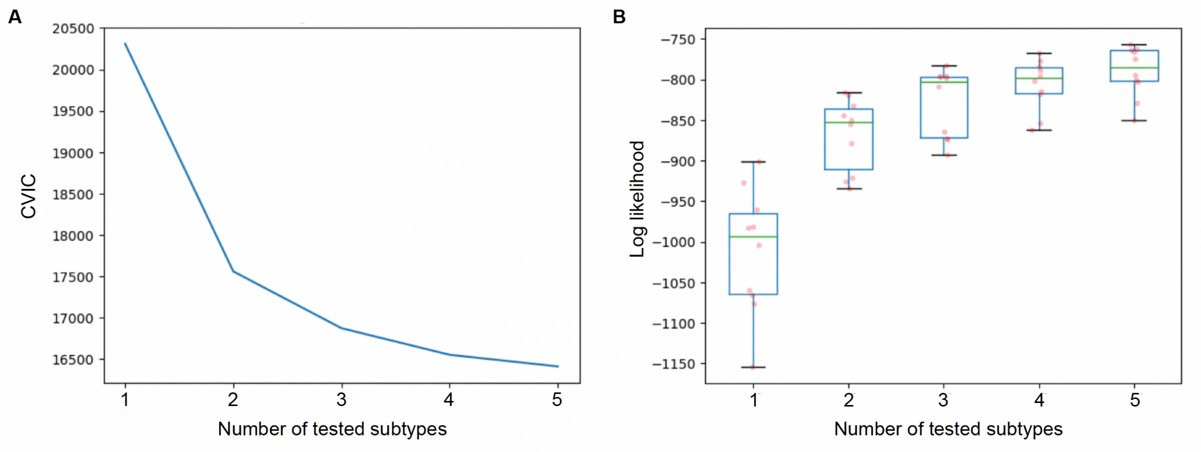

Model fit for SuStaIn models with varying number of subtypes. **A** Cross-validation information criterion (CVIC) and **B** log-likelihood across 10-folds of cross-validation are shown for 1-5 subtypes. Substantial improved model fit can be appreciated from one to two and two to three subtypes, but not for more subtypes. Therefore, three subtypes were selected.

Figure S2. Output of the SuStaIn model applied to complete data only (*N*=701)

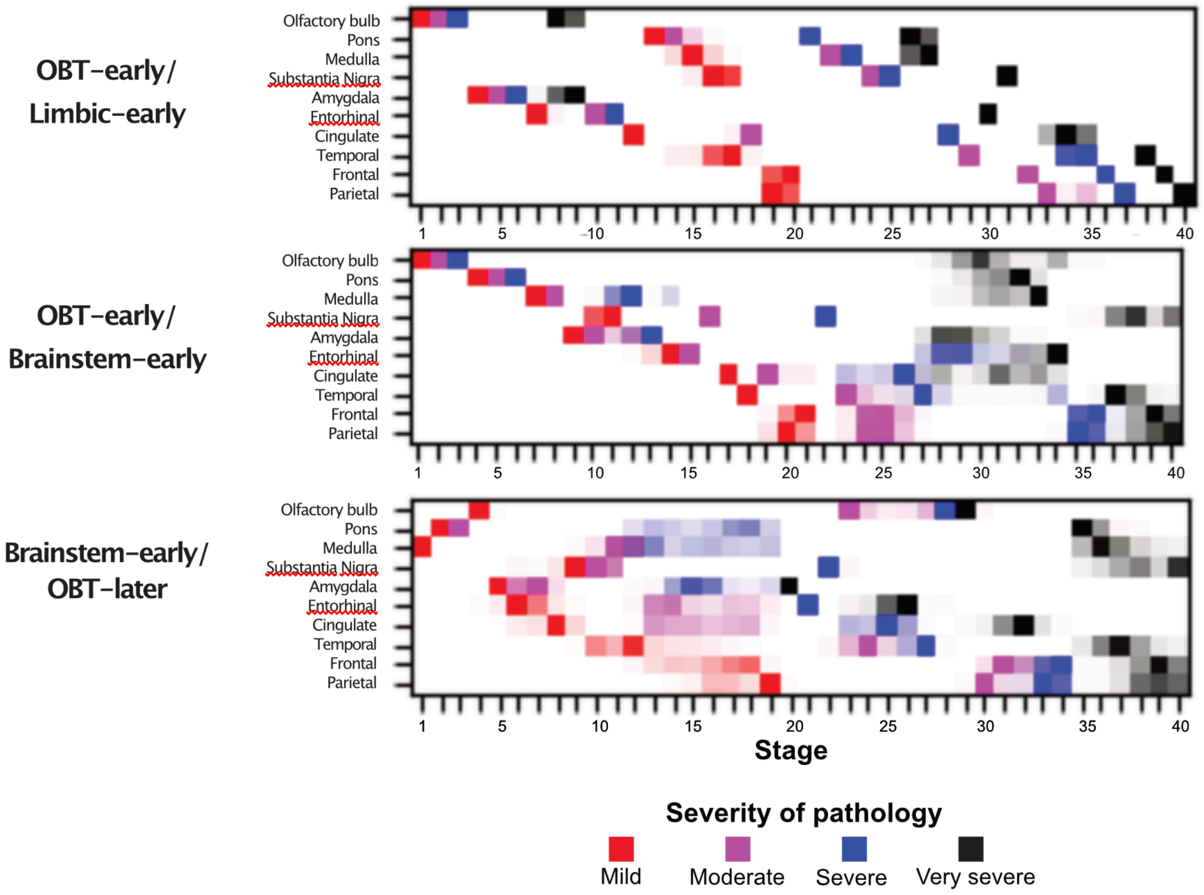

SuStaIn-inferred disease progression patterns of regional Lewy-type α-synuclein pathology in a subset of the BBDP cohort with complete *post-mortem* α-synuclein data (i.e., assessed in all 10 sampled regions). SuStaIn identified 3 subtypes of which the positional variance diagrams are shown, with each box representing the certainty that a brain region has reached a certain level of pathology (red = mild, purple = moderate, blue = severe, black = very severe) at a given SuStaIn or disease progression stage. Darker colors represent more confidence. Trajectories similar to the 3 subtypes in the main sample were identified.

OBT = olfactory bulb and tract; SuStaIn = Subtype and Stage Inference

Figure S3. Output of the SuStaIn model applied to re-examined OBT data

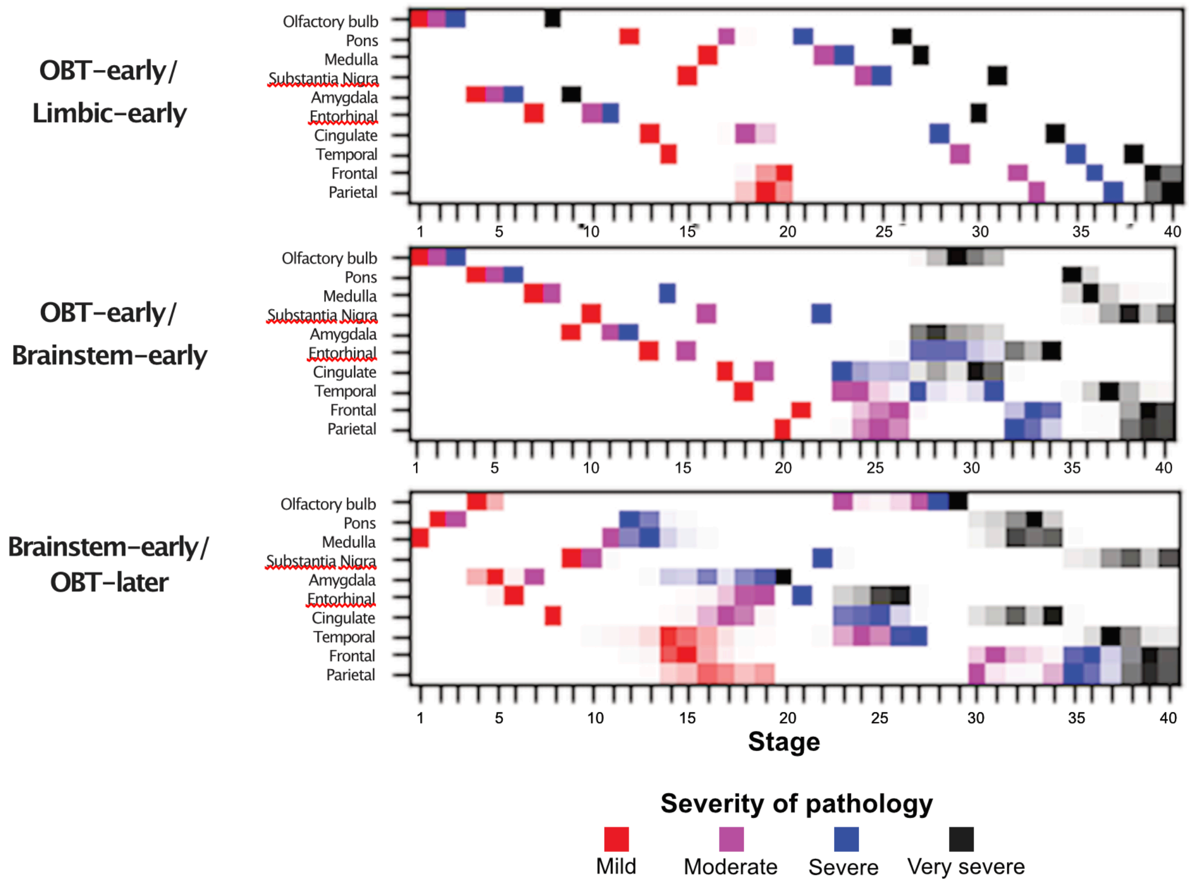

SuStaIn-inferred disease progression patterns of regional Lewy-type α-synuclein pathology in the BBDP cohort with re-examined OBT-stainings in OBT-negative cases of the Brainstem-early/OBT-later subtype. SuStaIn identified 3 subtypes of which the positional variance diagrams are shown, with each box representing the certainty that a brain region has reached a certain level of pathology (red = mild, purple = moderate, blue = severe, black = very severe) at a given SuStaIn or disease progression stage. Darker colors represent more confidence. Trajectories similar to the 3 subtypes in the main dataset were identified.

OBT = olfactory bulb and tract; SuStaIn = Subtype and Stage Inference

Figure S4. Subtype assignment probability across assigned stage

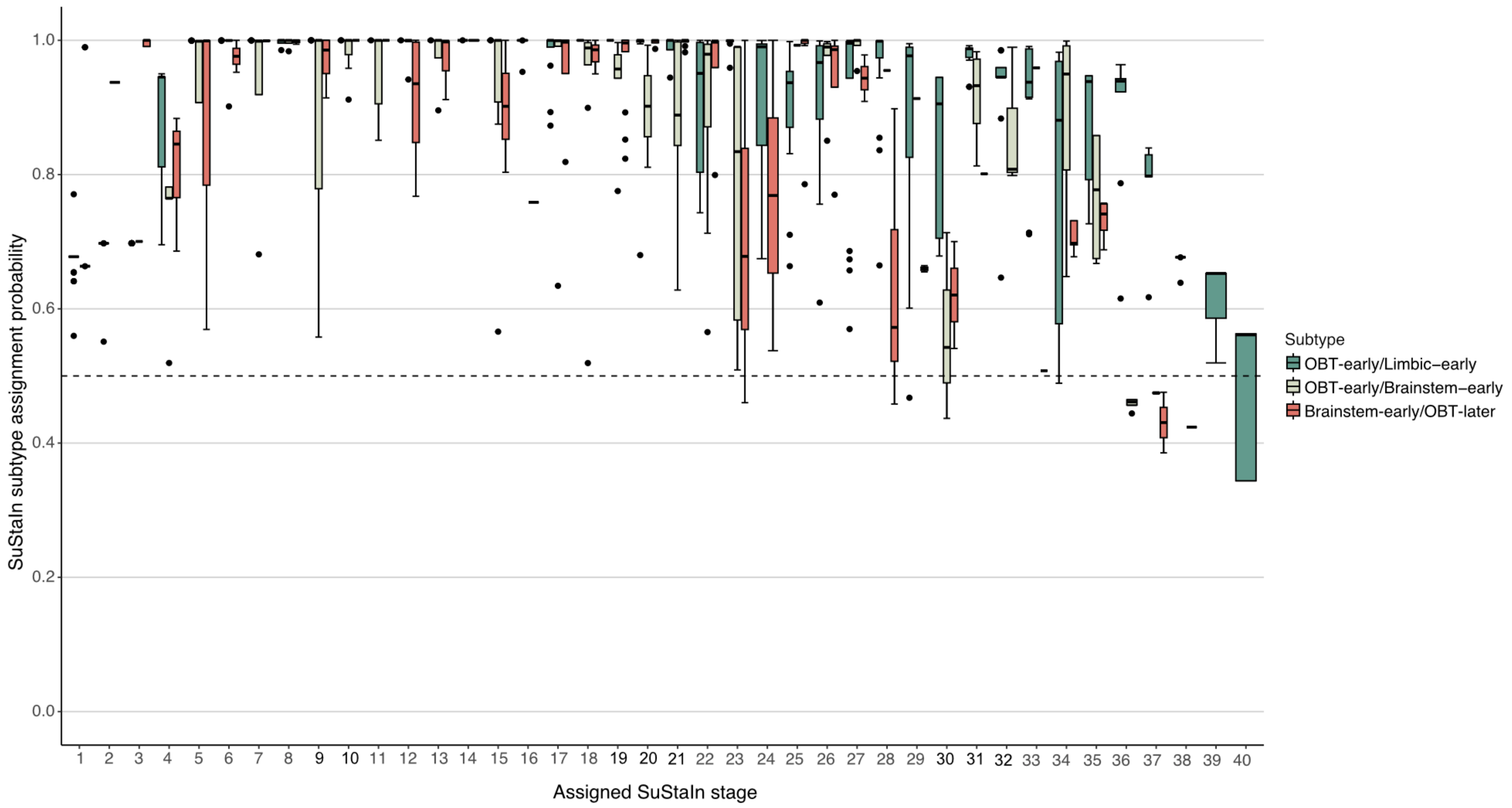

Boxplots show the probability of subtype assignment across assigned stages for the SuStaIn modelling cohort with stage>0 (*n*=801). The dashed line represents the cut-off of 50% for high probability. Subtype probability decreases with more advanced stages, reflecting similarity between subtypes. Boxplots show the median, lower, and upper quartiles with whiskers representing minimum and maximum values.

OBT = olfactory bulb and tract; SuStaIn = Subtype and Stage Inference

Figure S5. Distribution of stage assignment

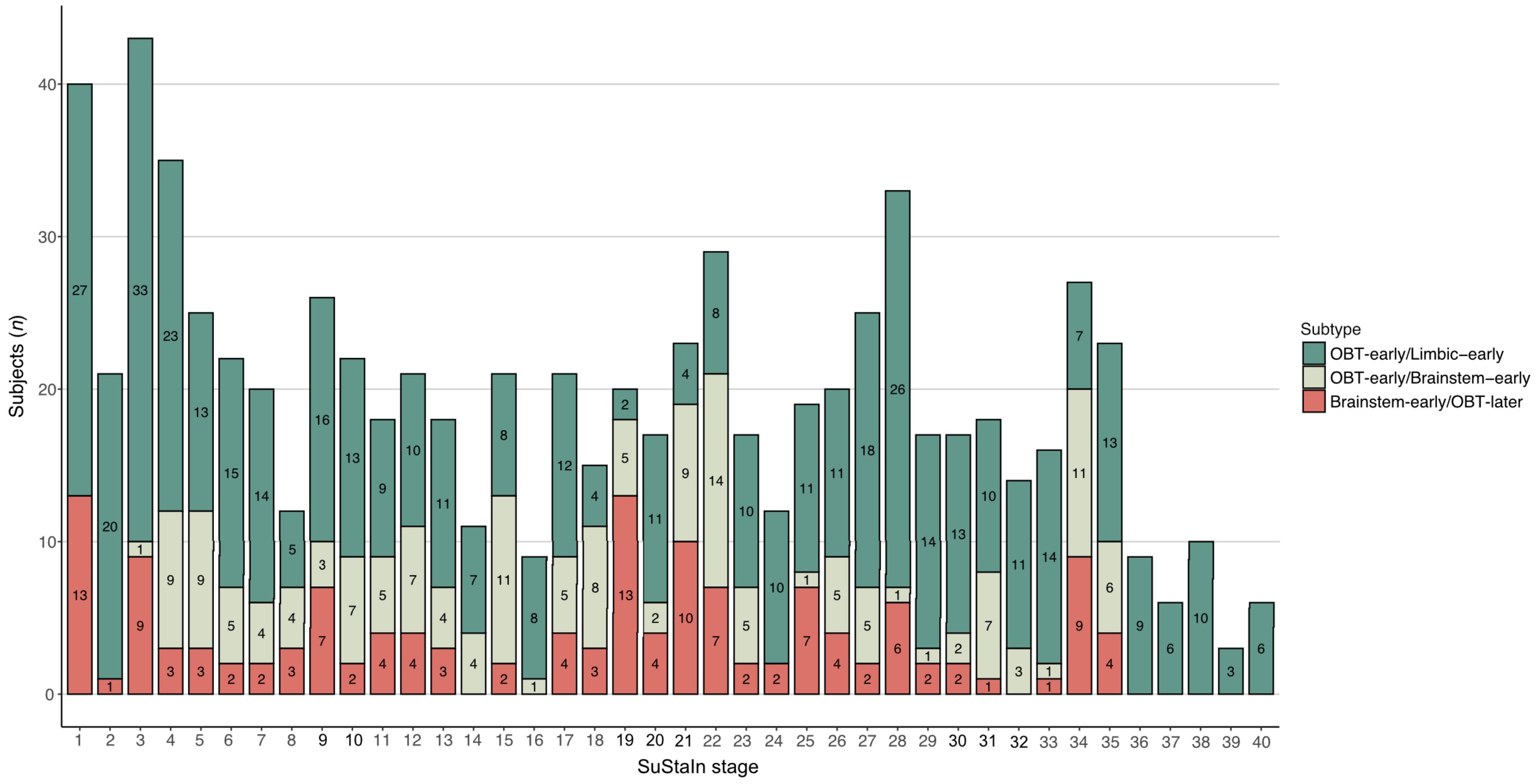

Bar chart shows the number of cases (stage>0, subtype probability>50%, *n*=781) assigned to each SuStaIn stage.
OBT = olfactory bulb and tract; SuStaIn = Subtype and Stage Inference

Figure S6. Number of subjects in stages of the Unified Staging Scheme for Lewy Body disorders according to SuStaIn stage

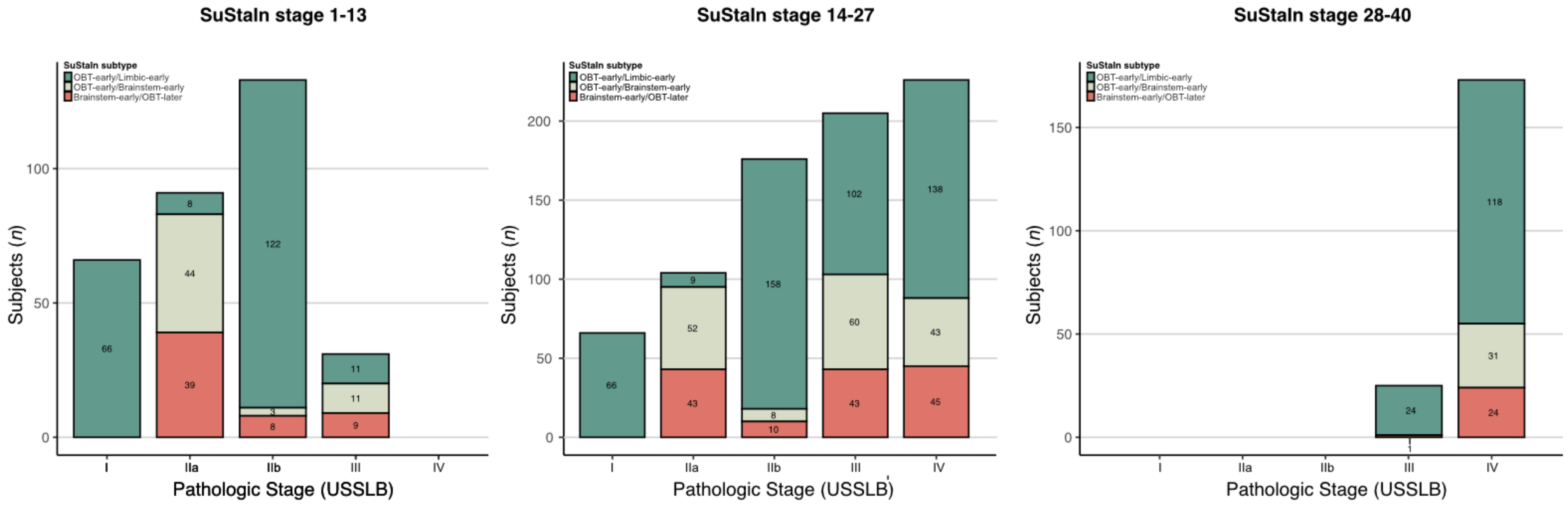

Staging of subjects (*n*=777) according to the Unified Staging Scheme for Lewy Body Disorders (USSLB), colored by SuStaIn subtype. Separate figures are shown for early (1-13), middle (14-27), and late (28-40) SuStaIn stage.

OBT = olfactory bulb and tract; SuStaIn = Subtype and Stage Inference

Figure S7. Depigmented neurons in the substantia nigra and GBA mutation status across LB subtypes

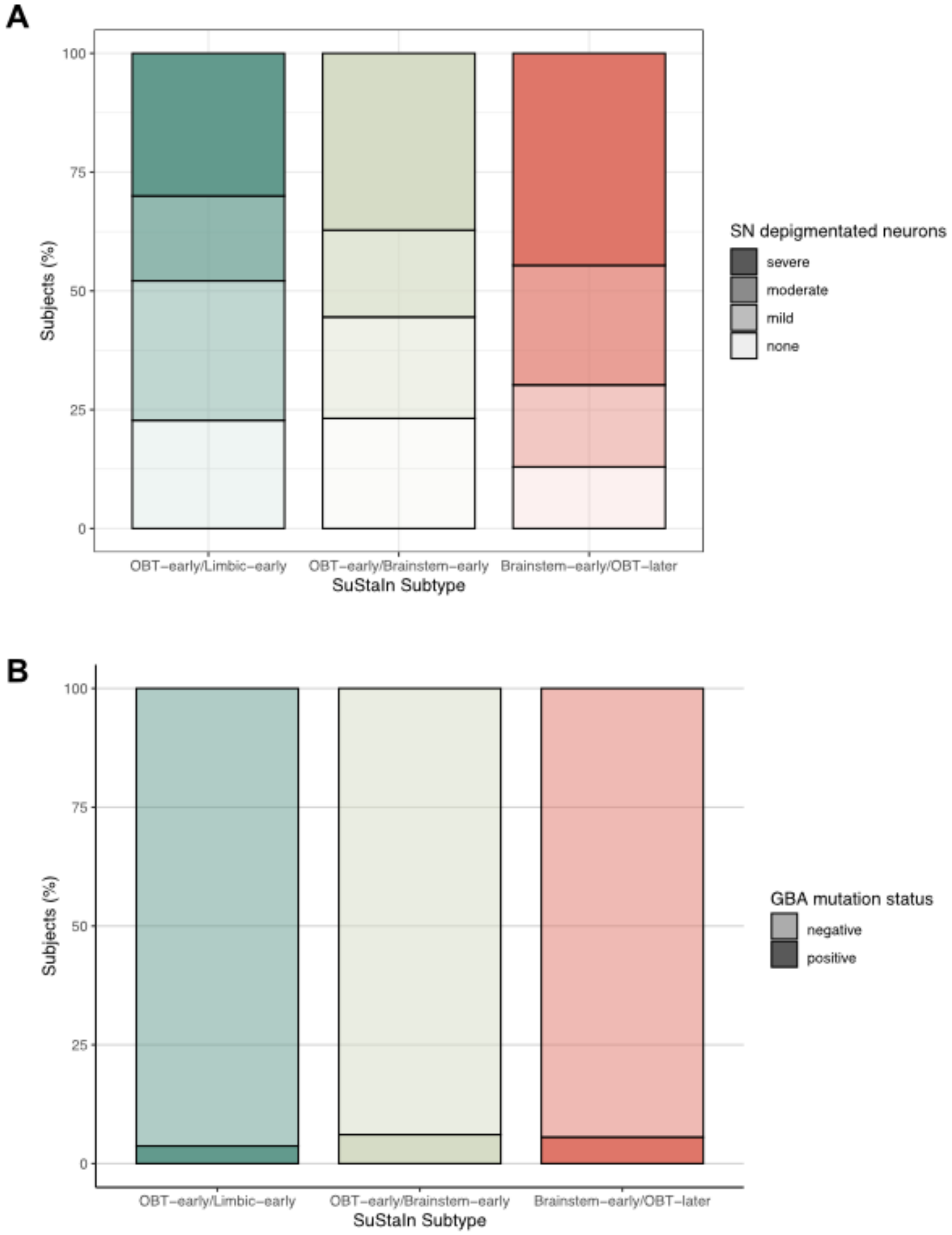

Subtypes compared on depigmented neurons and glucocerebrosidase (GBA) mutation status. **(A)** Depigmented neurons in the substantia nigra (SN) classified as none, mild, moderate, or severe (n=773). Analysis were performed with a multinomial regression model (S3 vs S1: SN level 2: p=0.001, SN level 3: p<0.001; S3 vs S2: SN level 3: p=0.003). **(B)** GBA nutation status (n=368). Comparisons are performed with logistic regression.

Figure S8. Sliding window analysis of LB subtype comparisons

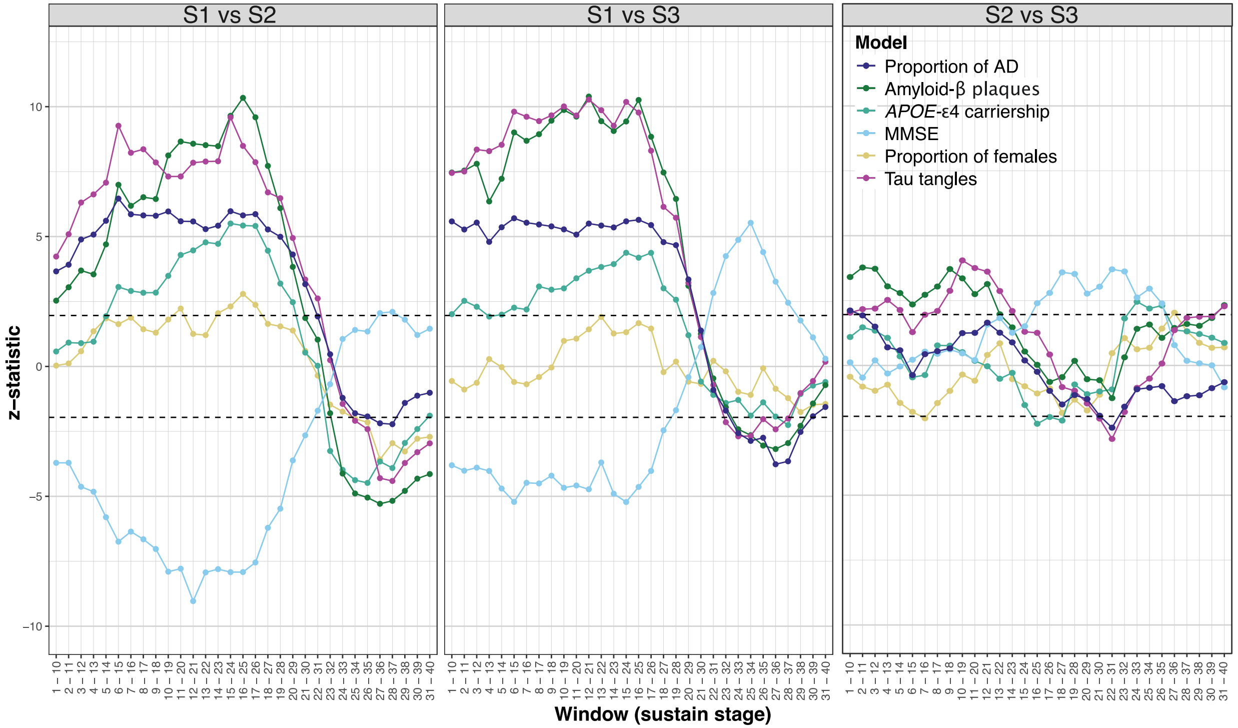

Subtype sizes for each window

Subtype	1–10	2–11	3–12	4-13	5-14	6-15	7-16	8-17	9-18	10-19	11-20	12-21	13-22	14-23	15-24
S1	150	139	132	108	95	93	85	79	82	78	69	62	63	56	57
S2	32	35	42	46	44	39	38	45	42	44	45	45	40	45	55
S3	36	30	31	26	27	27	25	25	22	19	20	29	29	36	43

Subtype	16-25	17-26	18-27	19-28	20-29	21-30	22-31	23-32	24-33	25-34	26-35	27-36	28-37	29-38	30-39	31-40
S1	59	61	60	67	83	98	108	113	113	114	117	113	108	91	83	80
S2	49	48	44	41	41	40	32	20	22	25	25	31	32	31	30	28
S3	43	45	48	49	38	40	32	27	26	24	18	23	25	19	17	15

Sliding window analysis (window width = 10, slide = 1) of subtype comparisons on the proportion of subjects with clinicopathological Alzheimer's disease, plaque burden, *APOE*-ε4 carriership, MMSE scores, the proportion of female subjects, and neurofibrillary burden. Each datapoint represents the test-statistic (*t*-value in case of continuous outcome variable and *z*-statistic in case of categorical outcome variable) of regression models adjusted for age, sex, and SuStaIn stage investigating subtype differences in a specific SuStaIn stage interval. Sample sizes across windows are shown in the table. AD = Alzheimer's disease; MMSE = Mini Mental State Examination; S1 = OBT-early/Limbic-early; S2 = OBT-early/Brainstem-early; S3 = Brainstem-early/OBT-later.

Figure S9. Total body Lewy body pathology across subtypes with a SuStaIn stage<10

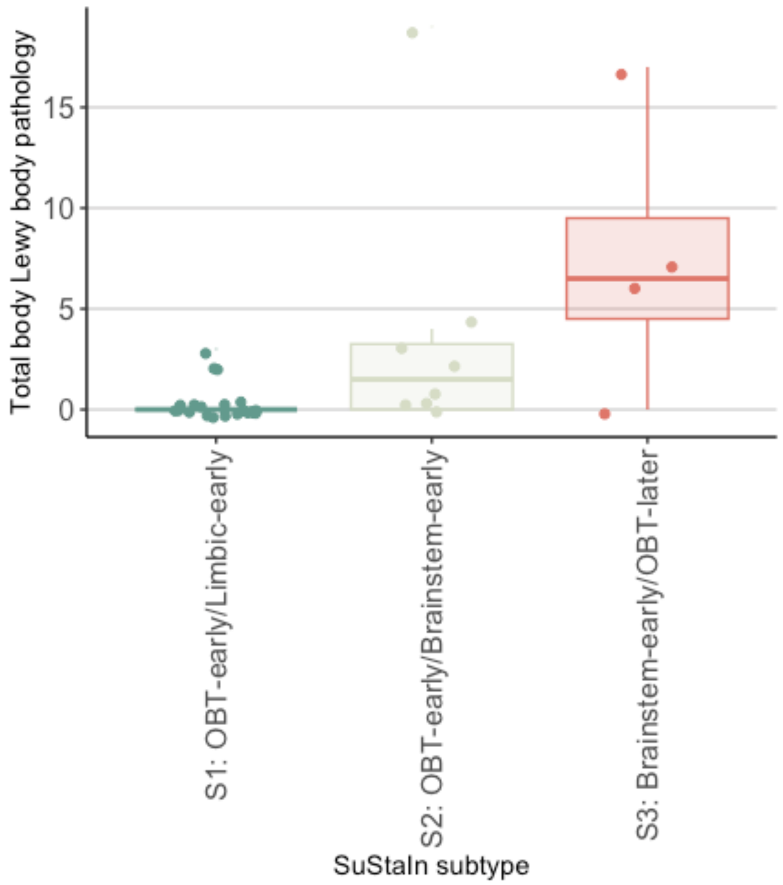

Total body Lewy body pathology in individuals in early SuStaIn stages (<10, *n*=36). Total pathology scores were computed as the sum of the α-synuclein density scores (0-4) assessed in the cervical, thoracic, lumbar, and sacral spinal cord gray matter, vagus nerve, submandibular gland, and esophagus, with higher scores indicating more severe pathology.
OBT = olfactory bulb and tract

Figure S10. Example images of various LTS density scores in the olfactory bulb

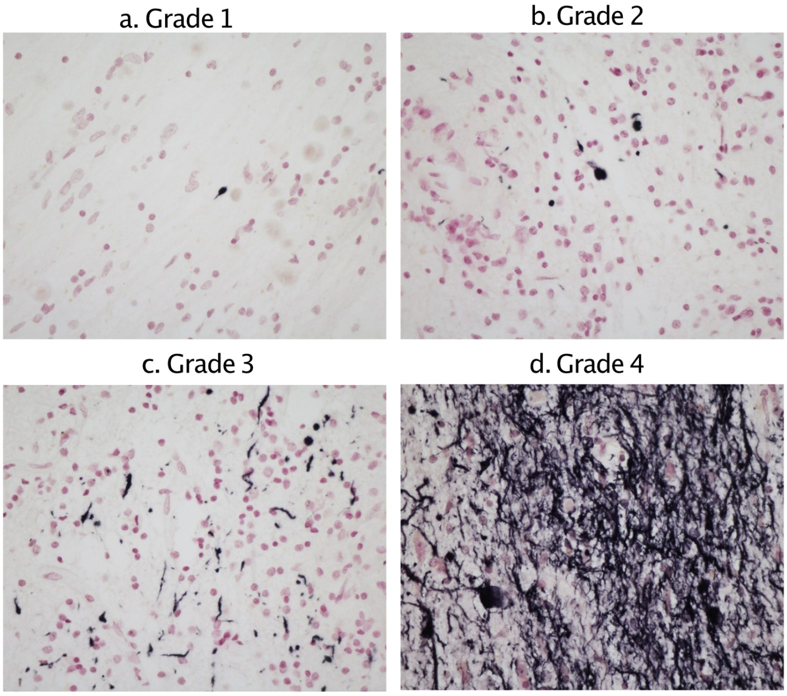

Photomicrographs of the immunohistochemical staining for α-synuclein in the olfactory bulb. Positive immunostaining is shown in black; the counterstain is Neutral Red. **A.** Mild pathology. **B.** Moderate pathology**. C.** Severe pathology**. D.** Very severe pathology**.**
